# Larvicidal proficiency of volatile compounds present in *Commiphora wightii* gum extract against *Aedes aegypti* (Linnaeus, 1762)

**DOI:** 10.3389/fpls.2023.1220339

**Published:** 2023-08-30

**Authors:** Krupal Patel, Divya Akbari, Rohan V. Pandya, Jigneshkumar Trivedi, Vishal Mevada, Shivraj Gangadhar Wanale, Rajesh Patel, Virendra Kumar Yadav, Jigna G. Tank, Dipak Kumar Sahoo, Ashish Patel

**Affiliations:** ^1^ Marine Biodiversity and Ecology Laboratory, Department of Zoology, Faculty of Science, The Maharaja Sayajirao University of Baroda, Vadodara, Gujarat, India; ^2^ University Grants Commission-Career Advancement Scheme (UGC-CAS) Department of Biosciences, Saurashtra University, Rajkot, Gujarat, India; ^3^ Department of Microbiology, Atmiya University, Rajkot, Gujarat, India; ^4^ Department of Life Sciences, Hemchandracharya North Gujarat University, Patan, Gujarat, India; ^5^ DNA Division, Directorate of Forensic Science, Gandhinagar, India; ^6^ School of Chemical Sciences, Swami Ramanand Teerth Marathwada University, Nanded, Maharashtra, India; ^7^ Department of Biosciences, Veer Narmad South Gujarat University, Surat, India; ^8^ Department of Veterinary Clinical Sciences, College of Veterinary Medicine, Iowa State University, Ames, IA, United States

**Keywords:** *Aedes aegypti*, crude extracts, larvicidal activity, volatile compounds, plant gum extract, viral vector

## Abstract

*Aedes* mosquitoes are the major cause of several vector-borne diseases in tropical and subtropical regions. Synthetic pesticides against these mosquitoes have certain limitations; hence, natural, eco-friendly, and safe larvicides obtained from plant resources are used to overcome these. In the present study, the larvicidal efficiency of *Commiphora wightii* against the fourth instar stage of the dengue fever mosquito *Aedes aegypti* (Linnaeus, 1762) was studied. The gum resin of *C. wightii* was collected using the borehole tapping method, and hexane extracts in different concentrations were prepared. The fourth-instar larvae were exposed to the extracts, and percent mortality, as well as LC_20_, LC_50_, and LC_90_, was calculated. Volatile compounds of the hexane gum extract were analyzed by Headspace GC/MS, and the sequence of the acetylcholine, Gamma-aminobutyric acid (GABA) receptor, and octopamine receptor subunit of *A. aegypti* was obtained. It was found that the hexane gum extract was toxic and lethal for larvae at different concentrations. Minimum mortality was observed at 164 µg mL^−1^ (10%/h), while maximum mortality was at 276 µg mL^−1^ (50%/h). The lethal concentrations LC_20_, LC_50_, and LC_90_ were 197.38 µg mL^−1^, 294.13 µg mL^−1^, and 540.15 µg mL^−1^, respectively. The GC/MS analysis confirmed the presence of diterpenes, monoterpenes, monoterpene alcohol, and sesquiterpenes in the gum samples, which are lethal for larvae due to their inhibitory activity on the acetylcholinesterase enzyme, GABA receptor, and octopamine receptor subunit. The use of commonly occurring plant gum for the control of mosquitoes was explored, and it was found that the gum of *C. wightii* had larvicidal activities and could be potentially insecticidal.

## Introduction

Mosquitoes are a well-known insect vector, transmitting various diseases that lead to millions of deaths every year (Acock et al., 2022; Kaavya et al., 2022). Globally, there is a documented presence of approximately 3,540 species (41 genera) of mosquitoes (Harbach and Besansky, 2014). Among these, *Aedes* mosquitoes are the main cause of viral vector-borne diseases such as chikungunya, Zika virus infection, yellow fever, hemorrhagic fever, and dengue fever in the tropical and subtropical regions (Yang et al., 2009; Kaavya et al., 2022). Out of these, dengue is a dominant and rapidly spreading disease caused by *Aedes aegypti* mosquitoes (Guzman and Harris, 2015). The transmission of dengue fever is facilitated by female *A. aegypti* mosquitoes, which deposit their eggs in stagnant water to raise the immature stages of their biphasic life cycle (Benelli and Mehlhorn, 2016).

Presently, the absence of efficacious dengue vaccines necessitates reliance on mosquito control as the sole preventive measure against the disease. This can be achieved through the wide use of different insecticides available. Synthetic larvicides are widely used in *Aedes* mosquitoes’ breeding places to control their vector-containing larva growth. However, deficiency in the control plans has resulted in the development of resistance by larvae against these synthetic larvicides (Braga et al., 2004; Medronho, 2008; Dusfour et al., 2011). In a study using WHO protocol to determine insecticide resistance and its mechanisms in the primary dengue vector in dengue-endemic districts of West Bengal, India, it was found that most *Aedes* populations were resistant to multiple synthetic insecticides (Bharati and Saha, 2018).

The development of insecticidal resistance has become a major issue in controlling vectors and, hence, vector-borne diseases. Such resistance can be induced due to changes in mosquitoes’ enzymatic system, leading to rapid detoxification and sequestration of exposed insecticide. Moreover, mutations in the target site can also prevent insecticide–target interaction (Hemingway et al., 2004). Synthetic larvicides also have an adverse effect on the environment.

In order to overcome these issues, many researchers have explored natural, eco-friendly, and safe larvicides obtained from plant resources. Various studies have documented biologically active plant compounds having efficient larvicidal and insecticidal effects on mosquitoes. It has been observed that these biologically active plant compounds are evolving and that they have no negative effects on the environment. They present the best alternative to synthetic pesticides, as they are environment-friendly, least toxic to human health, and convenient to use. Approximately 2,000 plant species are known for producing compounds that can be used for pest management, out of which 344 species are reported to be used against mosquitoes. Although these many species are known for mosquito control, phytochemical extracts of only a few plants have been known to act against mosquitoes in the form of growth regulators, repellents, and ovipositional barriers.

Guggulu is an oleo-gum resin that exudes from the bark of *Commiphora wightii* (Arnott) Bhandari or “Indian bdellium.” It has been used in Ayurveda since time immemorial to treat a variety of disorders, such as inflammation, gout, rheumatism, obesity, and lipid metabolism disorders (Sarup et al., 2015). *Commiphora wightii* resin incense was burned to repel mosquitoes traditionally by the tribes of Rajasthan, India (Kantheti and Padma, 2017).

The larvicidal activity of *C. wightii* gum resin and the effect of the identified volatile compounds on the development of *A. aegypti* larvae were analyzed. Moreover, the phytochemicals were screened against a homology model of the nervous system protein target acetylcholinesterase, the GABA receptor subunit, and the octopamine receptor of *A. aegypti* larvae.

## Materials and methods

### Collection of gum and preparation of hexane gum extract

The borehole tapping method was used to obtain gum resin from the bark of *C. wightii*. Pure hexane (Sigma Aldrich; purity: ≥99%; CAS Number: 592-41-6) was used to prepare the extract from the gum sample using the Soxhlet extraction procedure for 3 h (Vasishth and Guleria, 2017). Later, 200-mL samples were extracted and stored in a vacuum-tight glass reagent bottle at 4°C until further analysis.

### Culturing of *Aedes aegypti* larva

Dengue fever mosquitoes (*A. aegypti*) were cultured in the small mosquito insectary under laboratory conditions at the botanical garden of the Department of Biosciences, Saurashtra University, Rajkot, Gujarat, India. The colony was maintained in breeding cages 40 cm × 40 cm × 40 cm with standard conditions like temperature (28°C ± 1°C), humidity (80% ± 5% RH), and photoperiod (14L:10D) (Kumar et al., 2010; Udayanga et al., 2019). The fourth instar stage of *A. aegypti* larvae was used for bioassays.

### Larvicidal activity

The larvicidal activity was carried out at a laboratory scale. Different concentrations of the plant gum extracts in hexane were prepared to range from 164 µg mL^−1^ to 284 µg mL^−1^. For each hexane gum extract, 50 larvae of *A. aegypti* (fourth instar stage) were exposed for 60 min. The controls were exposed to the hexane solvent. The duration required for the mortality of half the number of larvae and all of the larvae in each concentration of hexane gum extract was recorded. Each bioassay was replicated in triplicates. The percent mortality of larvae was calculated using the following formula:


$$\text{Mortality}\;\left(\%\right)=\frac{\text{No. of dead larvae}}{\text{No. of total larvae}}\;\times \;100$$

### Statistical analysis

The percent mortality data were subjected to probit analysis for the calculation of LC_20_, LC_50_, and LC_90_. The regression analysis at the 95% confidence level and chi-square values were acculated by considering a *p*-value<0.05 to be statistically significant. The analyses were performed using Past software (4.03 version).

### Headspace GC/MS analysis of gum samples

The volatile compounds present in each hexane gum extract were analyzed by Headspace GC/MS. Their total ion chromatograms were obtained using a GERSTEL DHS System (Germany) connected to an Agilent 7890A GC and 5975C MS equipped with an Inert MSD with Triple Axis Detector (Germany). Each gum sample (1 g) was placed in a 20-mL standard headspace vial, which was placed in a tray of a GERSTEL Multipurpose Sampler. Volatile compounds were injected automatically into a CP-WAX 52 GC column (60 m × 25 μm film thickness × 0.25 mm) inner diameter (INNOWax, Germany). In the analytical conditions, the flow rate of helium gas was 1.2 mL/min, inlet pressure was 25 kPa, linear velocity was 1 mL/min at 210°C, injector temperature was 250°C, and injection mode was splitless. In the MS scan conditions, the source temperature was 200°C, the interface temperature was 250°C, the electron energy was 70 eV, and the mass scan was in the range of 40–350 amu. The temperature program was from 40°C to 240°C at a rate of 5°C/min. The relative percentage of each identified compound was calculated from the GC peak area. The compounds in each sample were identified through a comparison of spectral mass patterns and linear retention indices based on the n-alkane standards from C8–C32 with data reported in the Wiley and NIST databases. The retention index was calculated as per the retention index system proposed by Van Den Dool and Kratz (1963) using the following formula for temperature-programmed retention index:


$$\text{Ix}=100\left[\frac{\left(tx\;-tn\right)}{\left(t\left(n+1\right)-tn\right)}\;+n\right]$$

where *I_x_* is the temperature-programmed retention index, and *t_n_*, *t_n_*_+1_, and *t_x_* are the retention time (in min) of the two *n*-alkanes containing *n* and *n* + 1 carbons and the compound of interest, respectively.

### Homology modeling

The acetylcholinesterase and GABA receptor subunit protein and octopamine receptor sequences of *A. aegypti* were retrieved in FASTA format from the National Center for Biotechnology Information (https://www.ncbi.nlm.nih.gov/protein) with accession number AAB35001.1, AAA68961.1, and XP021692997.1, respectively. The protein’s three-dimensional structure was computationally generated with the homology modeling technique as described by the developer (Waterhouse et al., 2018).

### Molecular docking study

All molecular structures of phytochemicals reported in various fractions during LC-MS analysis were acquired from the PubChem database (https://pubchem.ncbi.nlm.nih.gov). The ligand library included natural substrates and established inhibitors. The protein structures and ligands were transformed to pdbqt format using Openbabel software, and the energy was minimized by implementing the Autodock Vina Tool’s Python script. Autodock Vina 1.2.0 was used for docking following the developers’ default parameters (Eberhardt et al., 2021). The software iGEMDOCK version 2.1 was employed to investigate postdocking ligand interactions (Hsu et al., 2011).

To model the target sequence, ProMod3 3.2.1 was used, and the SWISS-MODEL template library (SMTL version 2023-04-05, PDB release 2023-03-31) was searched for evolution-related structures that matched the target sequence.

The 6huj, 6xyy, and 2z73 PDB structures were used as a template for acetylcholinesterase, GABA receptor subunit, and octopamine receptor, respectively. The obtained model had 94.74%, 93.29%, and 79.88% amino acid residue in the Ramachandran plot favored region.

## Results

### Larvicidal properties against *A. aegypti*

**Figure 1:**
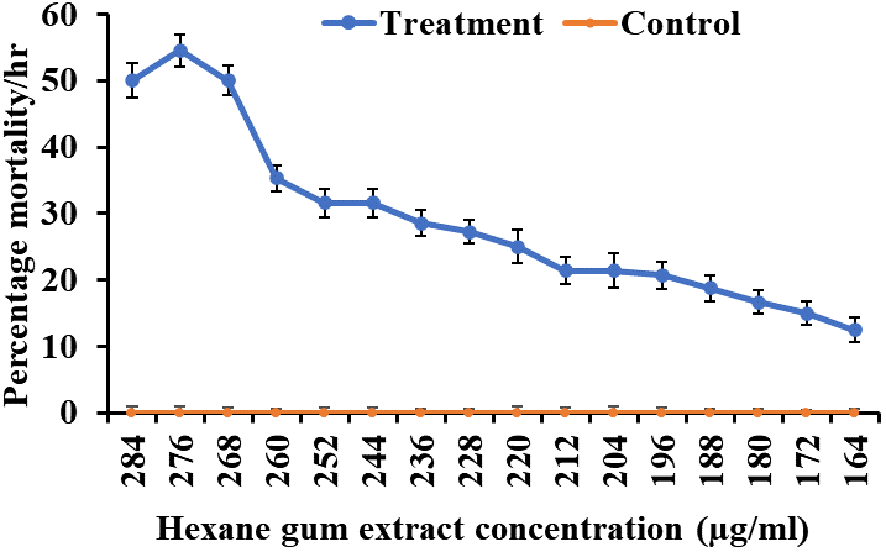
Mortality at different concentrations of *Commiphora wightii* gum hexane extracts against the fourth instar stage of *Aedes aegypti* larvae.

The larvicidal activity was examined in the range of 164–284 µg mL^−1^ (**Figure 1**). Minimum mortality was observed at the concentration of 164 µg mL^−1^ (10%/h), while maximum mortality was observed at the concentration of 276 µg mL^−1^ (50%/h). The lethal concentrations LC_20_, LC_50_, and LC_90_ were 197.38 µg mL^−1^, 294.13 µg mL^−1^, and 540.15 µg mL^−1^, respectively (χ2 = 0.905, *p*< 0.001) (**Table 1**). There was no significant mortality observed in the control group treated with only hexane solvent (**Figure 1**).

**Table 1:** Mosquito larvicidal effectiveness of hexane gum extract against *Aedes aegypti* (95% confidence interval) (***p< 0.001).

Sr. No.	Hexane gum extract	LC_20_ in µg mL^−1^	LC_50_ in µg mL^−1^	LC_90_ in µg mL^−1^	Chi-square χ^2^
	*Commiphora wightii*	197.38***	294.13***	540.15***	0.905

### Identification of volatile compounds from plant gum extracts prepared in hexane

Headspace GC/MS analysis was conducted for the hexane gum extract of *C. wightii*, which revealed the following types of volatile compounds: monoterpenes [*α*-Pinene (11.725%), 2-Norpinene 3,6,6-trimethyl- (10.607%), *α*-Ocimene (2.309%), *α*-Myrcene (5.289%), Limonene (5.324%), *α*-Terpineol (1.056%), *β*-Acoradiene (2.262%), and Sesquisabinene (1.140%)], sesquiterpenes [Caryophyllene (6.128%), *β*-Elemene (2.381%), *α*-Curcumene (3.961%), Sesquithujene (1.693%), and *α*-Bisabolene (1.033%)], and diterpenes [Cembrene (1.757%) and Neocembrene (9.950%)] (**Supplementary Figure S1**; **Table 2**). The refraction index of all identified compounds was calculated as per the retention index system proposed by Van Den Dool and Kratz (1963). The calculated retention indices of each identified compound were compared with the retention indices available in the NIST Chemistry WebBook, SRD 69 (**Table 2**). Previous studies have reported the presence of phytoconstituent groups such as monoterpenoids, sesquiterpenoids, diterpenoids, and triterpenoids; steroids; flavonoids; guggultetrols; lignans; sugars; and amino acids in the resin of *C. wightii*. Specific phytochemicals, *viz*., monoterpene (Limonene, *α*-Terpineol, *α*-Pinene, and *α*-Myrcene) and diterpene (Cembrene), have been reported in the gum resin of *C. wightii* (Sarup et al., 2015; Chandran et al., 2020).

**Table 2:** Volatile compounds from the hexane extract of *Commiphora wightii* gum.

Type of volatile compound	Volatile compound	RT	Peak area %	RI (calculated)	RI (NIST webbook database)
**Diterpene**	Cembrene	21.129	1.757	1,941	1,930
	Neocembrene	21.518	9.950	1,954	1,960
**Monoterpene**	2-Norpinene, 3,6,6-trimethyl-	3.517	10.607	916	Not available in database
	*α*-Pinene	3.406	11.725	907	909
	Limonene	5.103	5.324	1,032	1,039
	Sesquisabinene	13.190	1.140	1,463	1,461
	*α*-Ocimene	4.097	2.309	809	805
	*α*-Myrcene	4.313	5.289	981	986
	*β*-Acoradiene	11.821	2.262	1,454	1,462
**Monoterpene alcohol**	*α*-Terpineol	7.860	1.056	1,184	1,172
**Sesquiterpene**	*β*-Elemene	10.630	2.381	1,362	1,366
	Caryophyllene	11.179	6.128	1,456	1,467
	*α*-Curcumene	12.228	3.961	1,460	1,469
	Sesquithujene	12.511	1.693	1,410	1,413
	*α*-Bisabolene	12.801	1.033	1,446	1,443

### Molecular docking study

#### Acetylcholinesterase molecular docking

**Figure 2:**
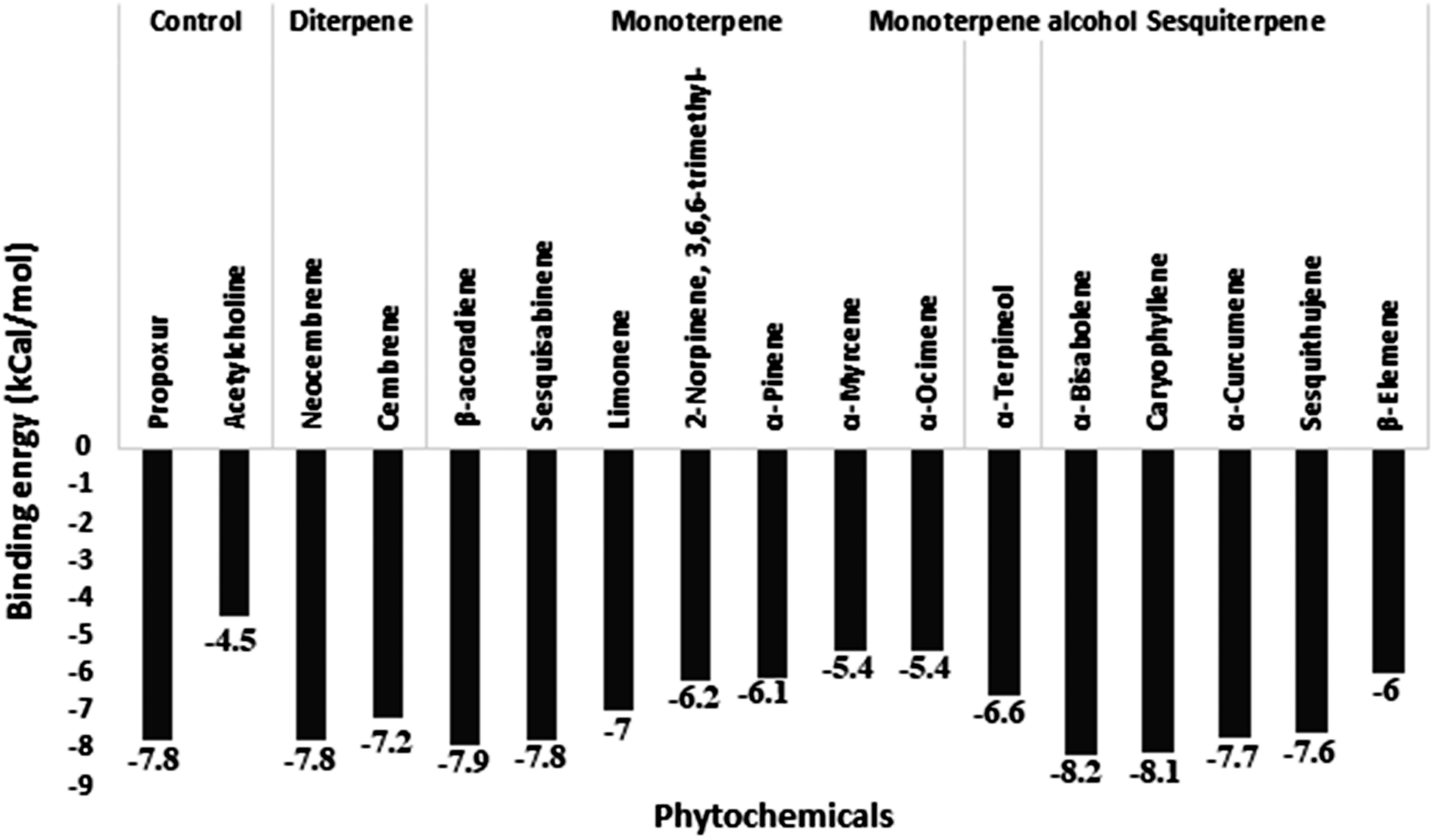
Molecular docking binding energy of Commiphora wightii phytochemicals against the acetylcholinesterase receptor.

**Figure 3:**
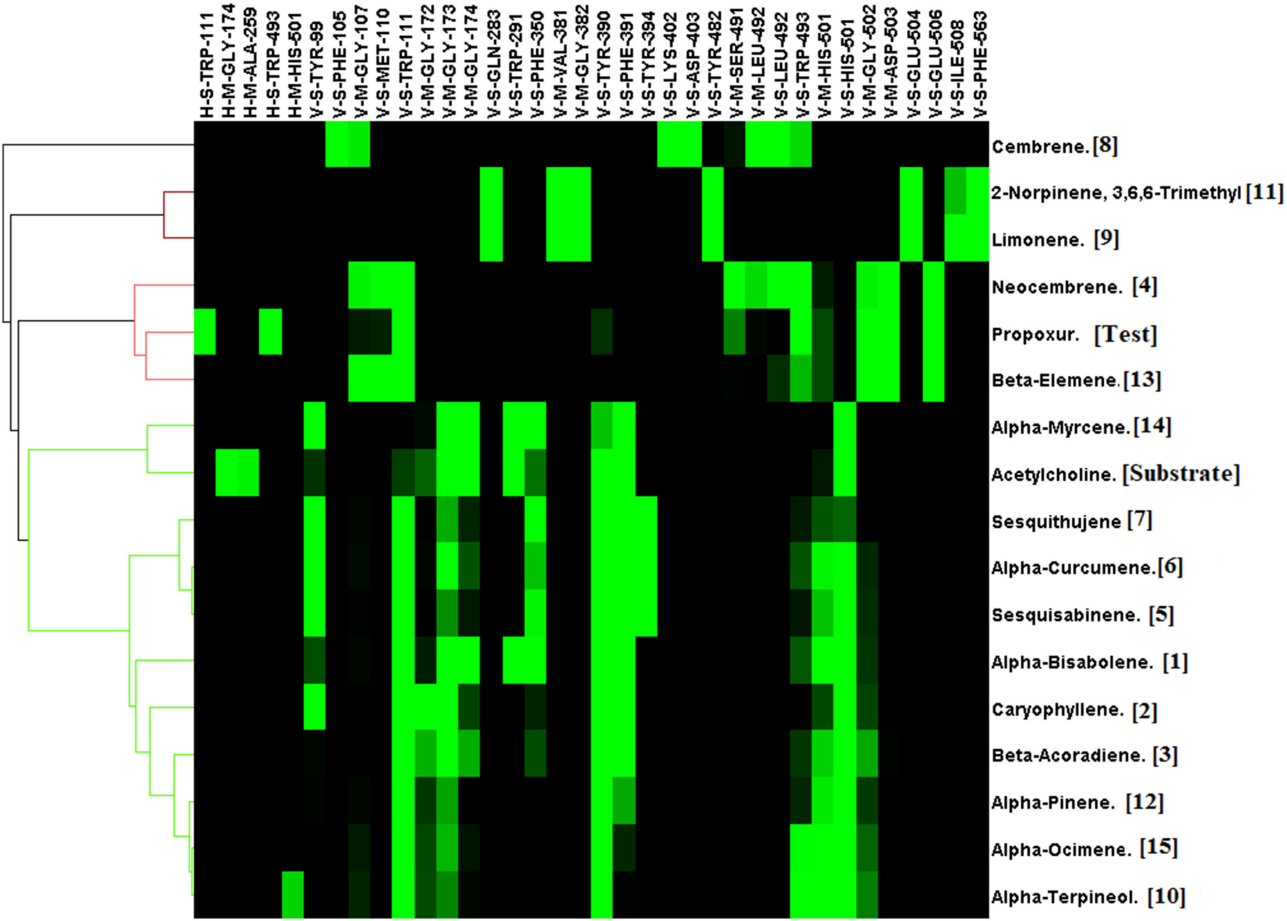
Interaction profile of *Commiphora wightii* phytochemicals best pose against the acetylcholinesterase receptor. Numbers in the bracket indicate the docking rank, black color indicates zero value, and green color indicates negative energy.

Acetylcholinesterase was docked against 15 unique phytochemicals, including the substrate acetylcholine and the inhibitor carbamate insecticide propoxur. The docking binding energy for phytochemicals ranged between -5.4 and -8.2. The binding energy of the substrate acetylcholine and the inhibitor carbamate insecticide propoxur was -4.5 and -7.8, respectively. The least binding energy was obtained for *α*-Bisabolene (-8.2), Caryophyllene (-8.1), and *β*-Acoradiene (-7.9), followed by other compounds (**Figure 2**). Acetylcholinesterase and docked phytochemical interaction analysis displayed diverse interaction profiles into five clusters. Acetylcholine and the inhibitor propoxur have been classified into different clusters. The three highest ranking *α*-Bisabolene, Caryophyllene, and *β*-Acoradiene had similar interaction profiles and shared a common bond pattern with substrate acetylcholine and inhibitor propoxur, indicating the potential of inhibition activity at the molecular level along with the obtained best docking score (**Figure 3**).

#### GABA receptor molecular docking

**Figure 4:**
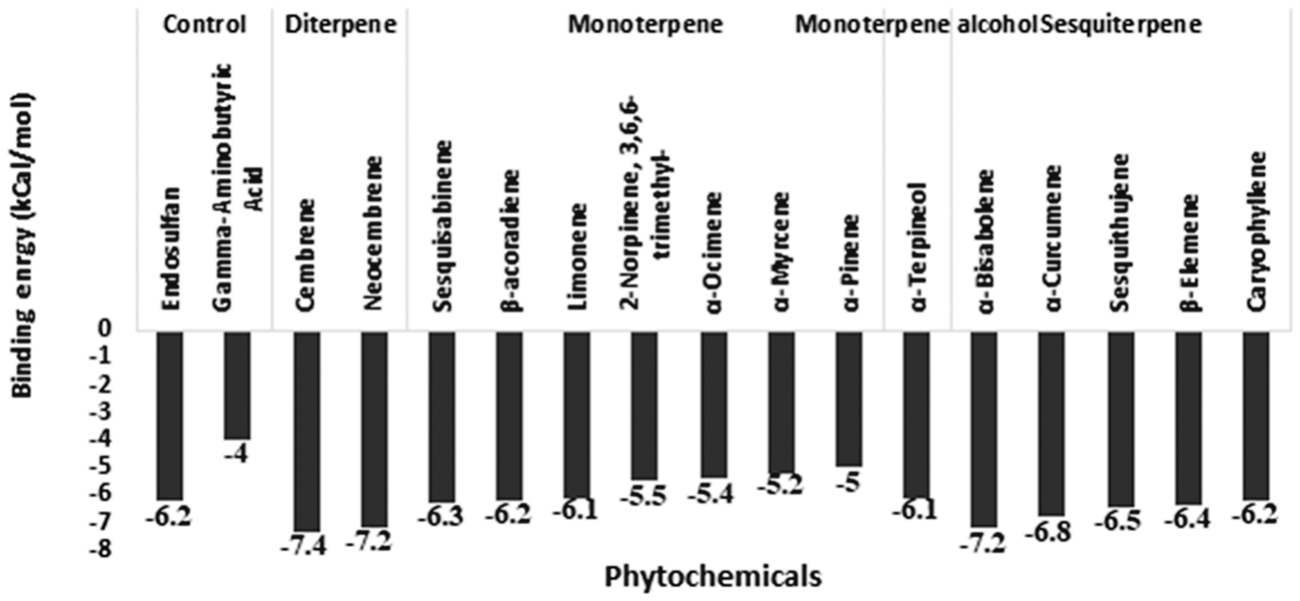
Molecular docking binding energy of *Commiphora wightii* phytochemicals against the GABA receptor.

**Figure 5:**
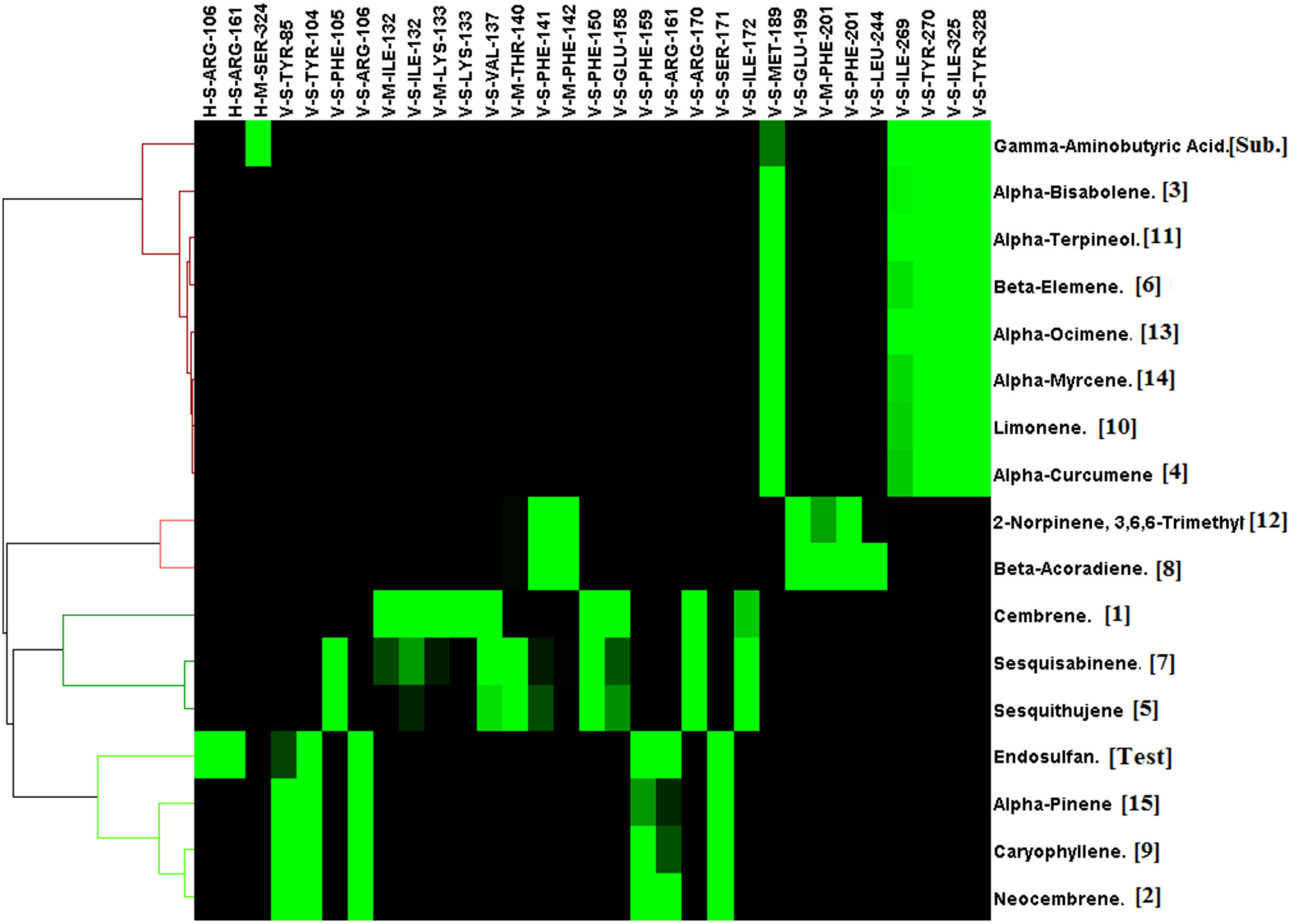
Interaction profile of *Commiphora wightii* phytochemicals best pose against the GABA receptor. Numbers in the bracket indicate the docking rank, black color indicates zero value, and green color indicates negative energy.

In addition to 15 phytochemicals for GABA receptors, natural substrate Gamma-Aminobutyric Acid and organochlorine insecticide endosulfan were also identified. The docking binding energy for phytochemicals ranged between -2.5 and -7.4. The minimum docking binding energy was obtained for Cembrene (-7.4), Bisabolene (-7.2), and Neocembrene (-7.2), followed by other compounds (**Figure 4**). The interaction analysis for GABA receptor molecular docking against phytochemicals revealed four distinct clusters (**Figure 5**). Substrate Gamma-Aminobutyric Acid was grouped in a unique cluster with a single member, and the inhibitor endosulfan was grouped in a different cluster with three other phytochemicals. Cembrene, the top-ranking compound, was also classified in a unique cluster with only one member, but it shared a few binding interactions with the inhibitor endosulfan. Conversely, Neocembrene, which is ranked second, pertains to the endosulfan cluster and exhibits a comparable binding interaction. On the contrary, the third compound with the highest rank, Bisabolene, exhibited a binding interaction that was comparable to that of Gamma-Aminobutyric Acid and was also found to be in close proximity (**Figure 5**).

### Octopamine receptor docking

**Figure 6:**
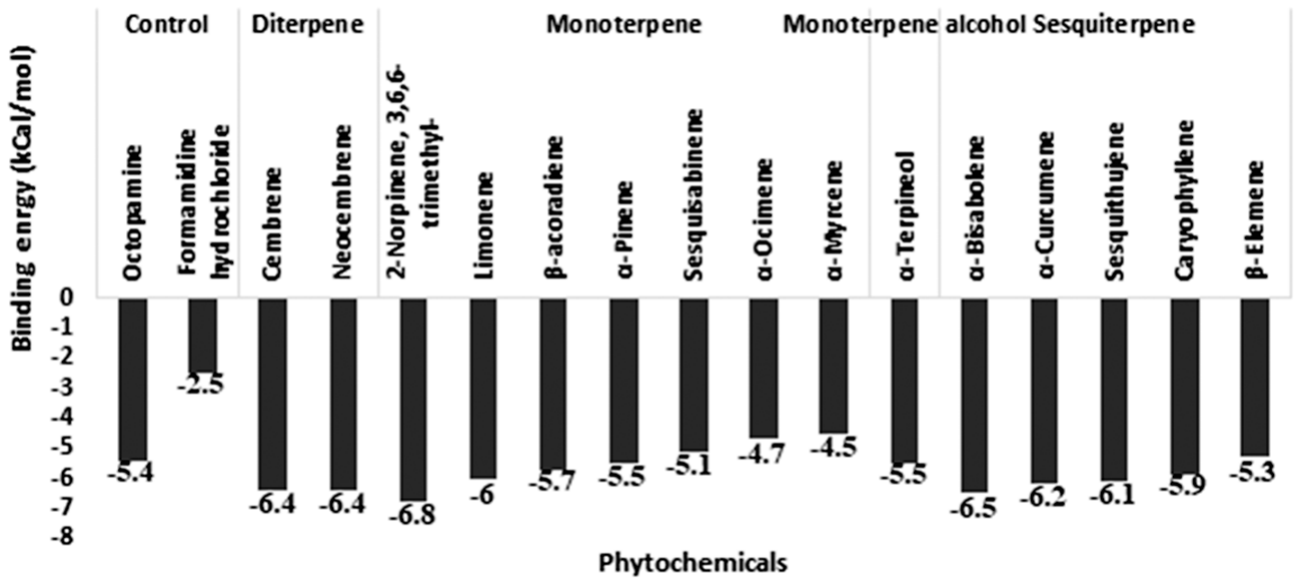
Molecular docking binding energy of *Commiphora wightii* phytochemicals against the octopamine receptor.

**Figure 7:**
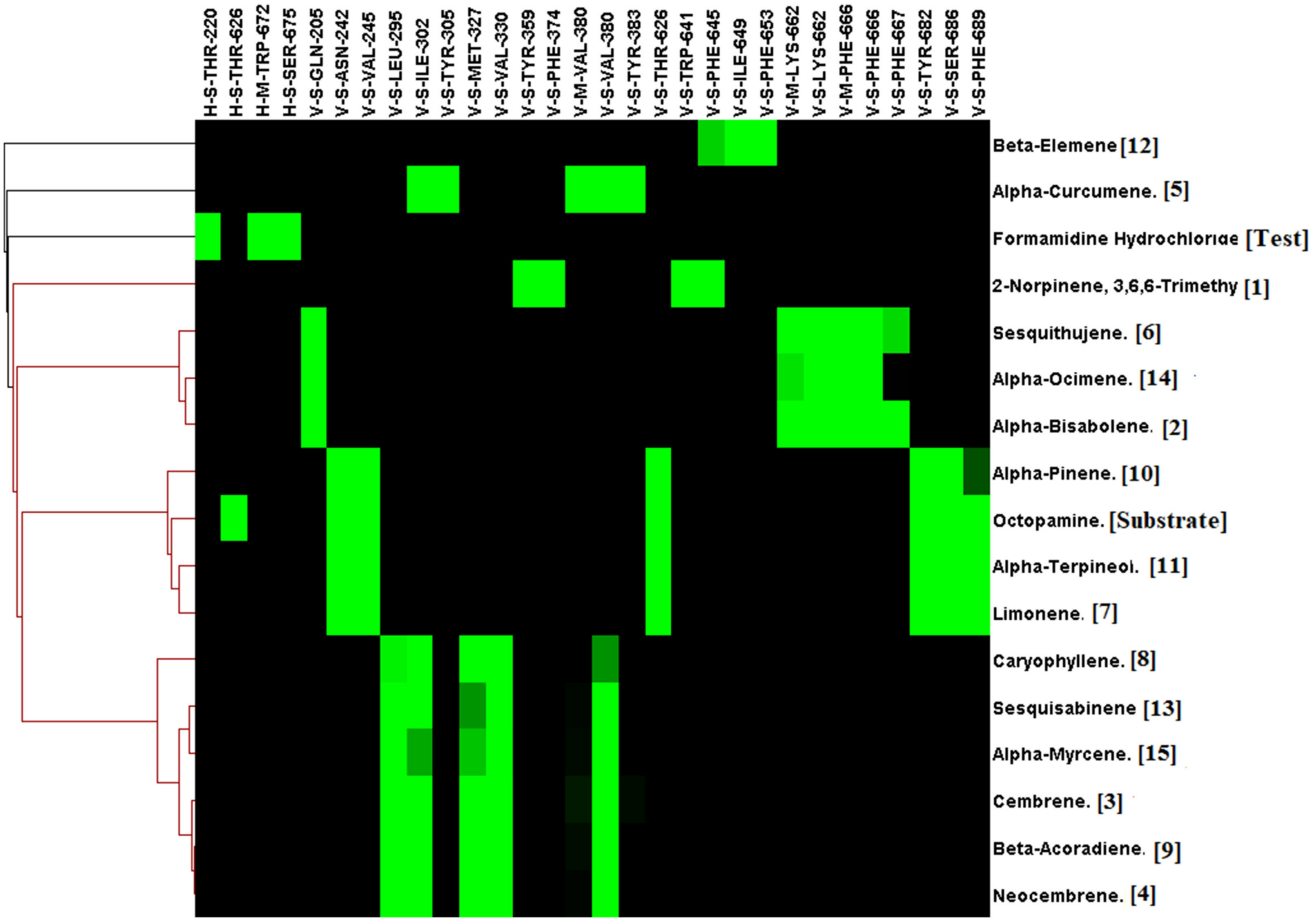
Interaction profile of *Commiphora wightii* phytochemicals best pose against the octopamine receptor. Numbers in the bracket indicate the docking rank, black color indicates zero value, and green color indicates negative energy.

For the octopamine receptor protein docking experiment, the binding energy ranged from –4.5 to -6.8 (**Figure 6**). The overall binding interaction for octopamine was relatively lower compared to that of acetylcholinesterase and GABA receptor, which may be due to the lower quality of the model. The minimum docking binding energy was obtained for 2-Norpinene, 3,6,6-trimethyl (-6.8), *α*-Bisabolene (-6.4), and Cembrene (-6.4), followed by other compounds (**Figure 6**). The interaction analysis for Octopamine receptor molecular docking against phytochemicals revealed four clusters, generated with a single member of with first-ranking 2-Norpinene, 3,6,6-trimethyl, *α*-Curcumene, *β*-Elemene, and inhibitor Formamidine hydrochloride. Second- and third-ranking compounds *α*-Bisabolene and Cembrene are also assorted in a separate group (**Figure 7**). In contrast, substrate octopamine was grouped together with the other three phytochemicals with a lower rank. The substrate octopamine had a lower binding energy of -5.4 when compared to the -2.5 of acaricide insecticide Formamidine hydrochloride.

Grouping of the three top-ranking compounds against generated leads the group of a total of six compounds, *α*-Bisabolene, Caryophyllene, Neocembrene, *β*-Acoradiene, 2-Norpinene, and 3,6,6-trimethyl that had better binding energy in comparison to their natural substrate and commercial inhibitor.

## Discussion

Since ancient times, extracts prepared from different parts of plants like flowers, bark, roots, leaves, and seeds have been used as insecticides (Sir et al., 2020). Various studies have reported the efficacy of plant extracts against mosquitoes. Studies have proven that these natural extracts have certain compounds that help in controlling the pest population (Jamiołkowska and Kopacki, 2020). These extracts have certain volatile compounds that can act on insects as insect repellents, insecticides, insect antifeedants, insect growth inhibitors, acetylcholinesterase enzyme inhibitors, oviposition inhibitors, ovicides, or insect growth-reducing agents (Hikal et al., 2017).

Numerous studies have reported the larvicidal activity of *Commiphora* species. The larvicidal activity of the following *Commiphora* species against different species of mosquitoes has been reported that includes oil-resins of *Commiphora molmol* (1,500 ppm acetone and aqueous extracts) against mosquito larvae of *Culex pipiens* (Baz et al., 2021); arabinofuranosidetridecanol from *C. merkeri* against *A. aegypti* (LC_50_ = 40.66  µg mL^−1^), *Anopheles gambiae* (LC_50_ = 22.86  µg mL^−1^), and *C. quinquefasciatus* (LC_50_ = 15.88  µg mL^−1^) (Samwel et al., 2021); essential oil of *C. erythraea* against *C. restuans*, *C. pipiens*, and *A. aegypti* (Muturi et al., 2020). As per the researchers, LC_50_<50 µg mL^−1^ is very active; LC_50_ 50 µg mL^−1^–100 µg mL^−1^ is active, and LC_50_ >100 µg mL^−1^ is a weak or inactive larvicidal compound (Milugo et al., 2021).

It has been suggested that monoterpenoids including Terpineol are very effective in inhibiting insect reproduction (Regnault-Roger et al., 2004), and the occurrence of monoterpenes and sesquiterpenes leads to fumigant toxicity (Hamraoui and Regnault-Roger, 1997; Regnault-Roger et al., 2004). Gum extracts are known as potential sources of larvicides due to their ability to alter the activity of the insect’s acetylcholinesterase enzyme (Ashokkumar et al., 2021), which is required for neuro-neuronal and neuromuscular junctions in insects. Among these, Limonene and *α*-Pinene are the most effective compounds that alter the activity of the acetylcholinesterase enzyme (Marei et al., 2012; Vieira et al., 2018).

Monoterpenes have natural pesticidal properties that are effective, safe, and biodegradable, which make them the best alternative to synthetic pesticides. Various pesticidal activities of monoterpenes, including bactericidal (Badawy et al., 2019), fungicidal, herbicidal (Liu et al., 2022), and insecticidal properties (Gad et al., 2022), are known. The larvicidal activity of monoterpenes against certain mosquito species (Gad et al., 2022), including toxic effects on *A. aegypti* (Giang An et al., 2020) and anti-oviposition effects on *A. aegypti* (Waliwitiya et al., 2009), has also been reported. It has been observed that monoterpenes and terpenoids are efficient acetylcholinesterase enzyme inhibitors in different insects and larvae (Waliwitiya et al., 2009), whereas diterpenes like Cembrene have antifeed activity against herbivores (Sun et al., 2019).

Previous research conducted on 24 species of the genus *Commiphora*, including *C. wightii*, identified 230 phytoconstituents, including phytoconstituents *α*-Bisabolene, Caryophyllene, Cembrene, Limonene, *α*-Pinene, *β*-Elemene, *α*-Myrcene, and *α*-Ocimene (Selvamani et al., 2008), which have also been obtained in the present study. However, Sesquisabinene and 2-Norpinene, 3,6,6-Trimethyl have not been reported from any *Commiphora* species so far.

The research conducted on five *Piper* species (*Piper nigrum*, *Piper mutabile*, *Piper longum*, *Piper montium*, and *Piper caninum*) showed excellent larvicidal activities with LC_50_ and LC_90_ values<10 μg mL^-1^ and were correlated with *β*-Caryophyllene, *β*-Bisabolene, *β*-Pinene, and *α*-Pinene concentrations (Huong et al., 2019), which support our molecular docking findings for *β*-Caryophyllene and *β*-Bisabolene.

*α*-Bisabolene was best docked against the acetylcholinesterase with binding energy −8.2. It has been observed that niloticin (−8.4 kcal/mol) had higher binding affinities and energy values than temephos (−4.75 kcal/mol), which is a commercial larvicide (Reegan et al., 2016). Similarly, the inhibitory effect of natural alkaloids on the acetylcholinesterase present in *A. aegypti* had the best fit into the AChE1 binding pocket with a minimum binding energy of −8.13 (Balachandran et al., 2021).

The gamma-aminobutyric acid receptor, also known as the GABA receptor, has four distinct but overlapping and coupled targets of pesticide action. These targets are associated with very little or no cross-resistance (Setlur et al., 2023). In the present research work, besides acetylcholinesterase, *α*-Bisabolene also ranked second and third for the octopamine receptor and GABA receptor, indicating its possibility to act as a multitarget ligand inhibiting the nervous system. Thus, *α*-Bisabolene emerged as one of the potent multitarget inhibitors for application as a bio-insecticide to control the resistant *A. aegypti.*

Bisabolene, a plant-based Sesquiterpene, is used as a precursor for the synthesis of several industrially relevant chemicals (Zhao et al., 2021). Bisabolene’s low plant abundance makes its isolation uneconomical. Therefore, industrial Bisabolene production using synthetic biology and metabolic engineering to create microbial cell factories is more competitive and environmentally sustainable. In this proof-of-principle engineering, the oleaginous yeast *Yarrowia lipolytica* was explored to produce *α*- and *β*-Bisabolene by heterologously expressing the synthase genes from *Abies grandis*, *Zingiber officinale*, and *Helianthus annuus*.

The current research findings suggest that *C. wightii* could be beneficial as a biological agent in the fight against *A. aegypti*. It was found that Bisabolene could be one of the key larvicidal compounds that employ a multitarget strategy and act on proteins associated with the nervous system of *A. aegypti* larvae. Even in cases where *A. aegypti* larva has developed resistance to commercially used pesticides, being a natural compound, Bisabolene can be effective against them.

## Conclusion

*A. aegypti* mosquito, also known as the yellow fever mosquito, is a potent transmitter of several vector-borne diseases such as dengue fever, chikungunya, Zika fever, the Mayaro virus, and yellow fever, in addition to other pathogens. Guggulu, also known as *C. wightii*, has recently been recognized as a reliable source of traditional medicines for the treatment of a variety of conditions, including inflammation, arthritis, obesity, microbial infection, wounds, pain, fractures, tumors, and gastrointestinal diseases. The current investigation posed an additional application of *C. wightii* gum resin for the environment-friendly biological control of *A. aegypti* larva (fourth instar) due to its multitarget action. A total of 15 volatile compounds were identified belonging to diterpene, monoterpene, monoterpene alcohol, and sesquiterpene groups. A total of 230 phytoconstituents have been identified from *Commiphora* species, out of which five unique compounds were identified from *C. wightii*, which were Limonene, *α*-Terpineol, *α*-Pinene, *α*-Myrcene, and Cembrene. The molecular docking study of acetylcholinesterase, GABA receptor, and octopamine receptor revealed certain compounds of the *C. wightii* gum resin having very low binding energy, revealing their role in larvicidal properties. These compounds are known to act upon the nervous system leading to the ultimate death of the larvae.

## Data availability statement

The datasets presented in this study can be found in online repositories. The names of the repository/repositories and accession number(s) can be found in the article/**Supplementary Material**.

## Ethics statement

The article presents research on animals that do not require ethical approval for their study.

## Author contributions

Conceptualization and supervision: AP, JGT, VY, and DS. Investigation and methodology: KP, DA, RVP, VM, and SW. Original draft preparation: AP, VY, KP, and JGT. Review and final editing: RP, AP, JGT, and JT. All authors contributed to the article and approved the submitted version.
