# Supplementary figures and images for: Larvicidal proficiency of volatile compounds present in Commiphora wightii gum extract against Aedes aegypti (Linnaeus, 1762)
Source: Front Plant Sci. 2023 Aug 30;14:1220339. doi: 10.3389/fpls.2023.1220339 (PMC10499046; doi:10.3389/fpls.2023.1220339)

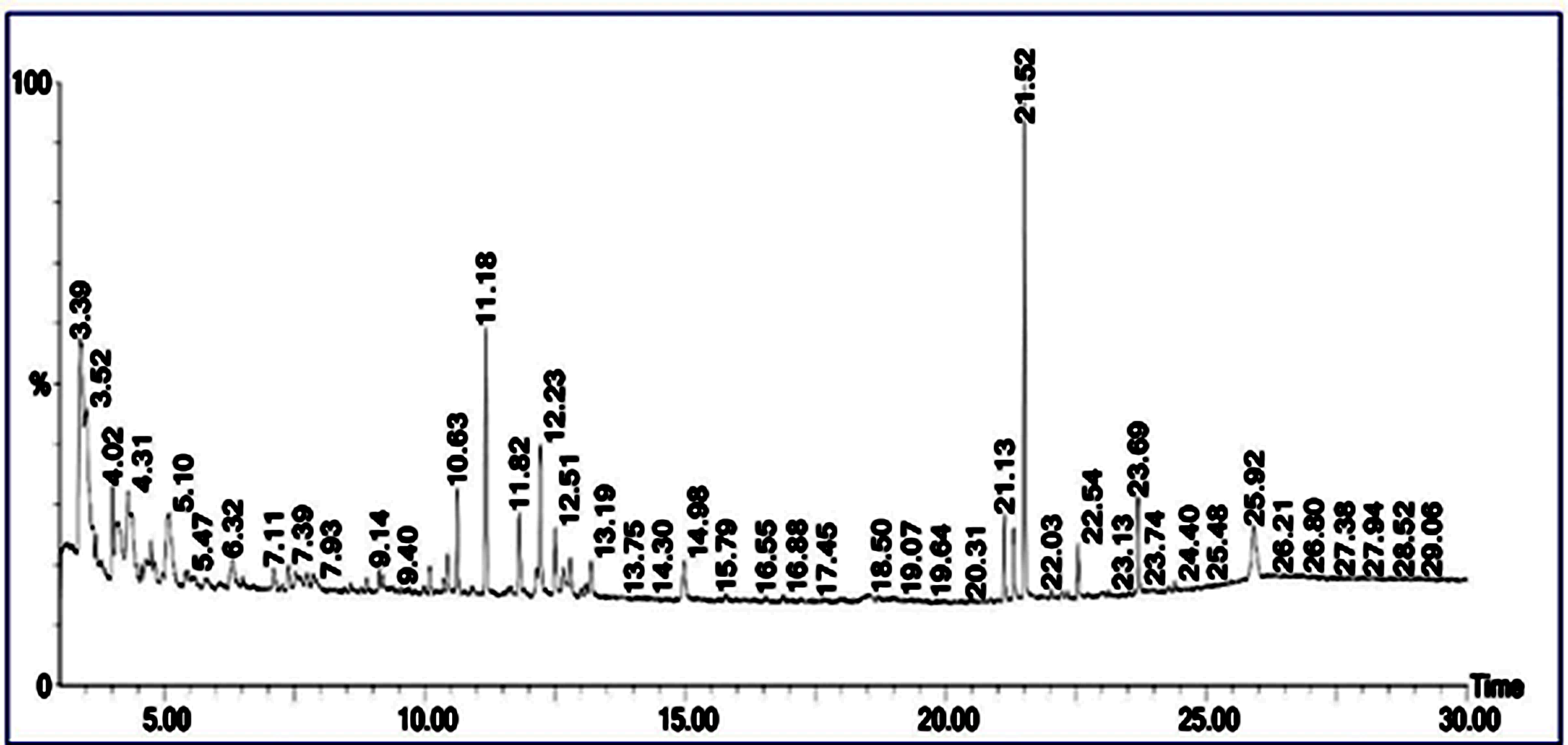

Supplement: Supplementary file 1 [file Image_1.tif]
